# Preferential lattice expansion of polypropylene in a trilayer polypropylene/polyethylene/polypropylene microporous separator in Li-ion batteries

**DOI:** 10.1038/s41598-021-81644-3

**Published:** 2021-01-21

**Authors:** Wen-Dung Hsu, Po-Wei Yang, Hung-Yuan Chen, Po-Hsien Wu, Pin-Chin Wu, Chih-Wei Hu, Lakshmanan Saravanan, Yen-Fa Liao, Yen-Teng Su, Dinesh Bhalothia, Tsan-Yao Chen, Chia-Chin Chang

**Affiliations:** 1 Department of Materials Science and Engineering, National Cheng Kung University, Tainan, 70101 Taiwan; 2 Hierarchical Green-Energy Materials Research Center, National Cheng Kung University, Tainan, 70101 Taiwan; 3 Department of Engineering and System Science, National Tsing-Hua University, Hsinchu, 300 Taiwan; 4 R & D Center for Li-Ion Battery, National University of Tainan, Tainan, 70005 Taiwan; 5 Department of Green Energy, National University of Tainan, Tainan, 70005 Taiwan; 6 National Synchrotron Radiation Research Center, 101 Hsin-Ann Road, Hsinchu, 30013 Taiwan; 7 BenQ Materials Corporation, Guishan, Taoyuan, 33341 Taiwan

**Keywords:** Energy science and technology, Materials science

## Abstract

The abnormal lattice expansion of commercial polypropylene (PP)/polyethylene (PE)/polypropylene (PP) separator in lithium-ion battery under different charging current densities was observed by in-situ X-ray diffraction. Significant lattice changes of both PP and PE were found during the low current density charging. The capacity fading and the resistance value of the cell measured at 0.025 C (5th retention, 92%) is unexpectedly larger than that at 1.0 C (5th retention, 97.3%) from the electrochemical impedance spectroscopic data. High-resolution scanning electron microscopy is employed to witness the pore changes of the trilayered membrane. Density functional theory calculations were used to investigate the mechanism responsible for the irregular results. The calculations revealed that the insertion of Li-ion and EC molecule into PP or PE are thermodynamically favourable process which might explain the anomalous significant lattice expansion during the low current density charging. Therefore, designing a new separator material with a more compact crystalline structure or surface modification to reduce the Li insertion during the battery operation is desirable.

## Introduction

Lithium-ion batteries (LIBs) is a key module in portable consumer electronics. In the recent era, the demand even increased intensively due to their wide applications in the industrial usage of electrical vehicle and power storage systems. Most of the studies working on Li-ion batteries that aiming at the applications in the utility storage or transportations focuses on developing low-cost electrode materials with high capacity, long cycle life and best safety^1^. On the other hand, separators, made up of polymer membranes, separating cathode and anode electrodes, acting as an electrolyte reservoir and assisting the transport of ions^2, 3^, can also strongly affects the cell performance. Particularly, their porous structure plays a vital role to determine battery performance. Most separators available in the market are porous polyolefin materials composed of polyethylene (PE)^4, 5^ and polypropylene (PP) membranes with thickness less than 25 μm^6–8^ due to their high stability and low cost. The multi-layer separator such as PP-PE-PP trilayer laminates has been patented as a high heat-resistant separator which provides adequate thermal and chemical stability and mechanical strength^2, 9–11^. Their properties such as crystallinity, porosity, thickness, electrolyte adsorption, chemical, mechanical and thermal stability etc., however, could be changed at long or extreme operating conditions such as severe temperatures. Although the polyolefin separators are effective for portable electronic devices, they suffer two major disadvantages. First, due to their intrinsic hydrophobicity and low surface energy, they provide low wettability and durability in liquid electrolytes which contain polar solvents. Last, due to their low melting points, the polyolefin separators suffer thermal shrinkage at elevated temperatures^10, 11^.

Porosity is a significant feature of a separator. A good separator should have proper pore size and should be distributed evenly to suppress lithium dendrite and prevent active particles from passing through the separators^12, 13^. By combining viscoelastic and poroelastic behaviour, Gor and his colleagues determined the behaviours of both dry and wet manufacture processes of separators under compressive loading^14^. Christina and Arnold^15^ claimed that the capacity fading was caused by a viscoelastic slink in the compressed separator which shrinks the pore size and impedes the ion transport resulting in increase of internal resistance. Love^10^ specified that the pore shrinkage was caused by applying the static and dynamic loading below the temperature of melting.

To our knowledge, the in-situ XRD studies on crystalline structural changes of the trilayer polymer separators in lithium-ion battery during charge/discharge cycle are rare. There are also very few studies that discussed the effect of very low charge/discharge (C/DC) current (0.025 C) on the separator of LIB system^16, 17^. The very low charge/discharge current occurs when the battery is used as back-up power for emergency light or other small sensors. Therefore, it is necessary to understand the effect of separator in those applications. In this work, the evolution of the crystalline structure of the commercial trilayered PP/PE/PP separator was investigated by in-situ X-ray diffraction technique during the LIB charging process. For the low-angle XRD analysis, a special coin cell was fabricated. The high-resolution scanning electron microscopy (HR-SEM) also witnessed the changes in this separator's microporous morphology at different charging current density. Rate performance and the columbic efficiency of the cell was measured after cycled for 5 C/DC cycles at the rate of 1.0 C and 0.025 C. The first-principles calculations were further applied with VASP implemented density functional theory (DFT) to analyse the ions and electrolytes insertion reactions in the trilayered separator.

## Results and discussion

### Electrochemical performance and EIS analyses

**Figure 1:**
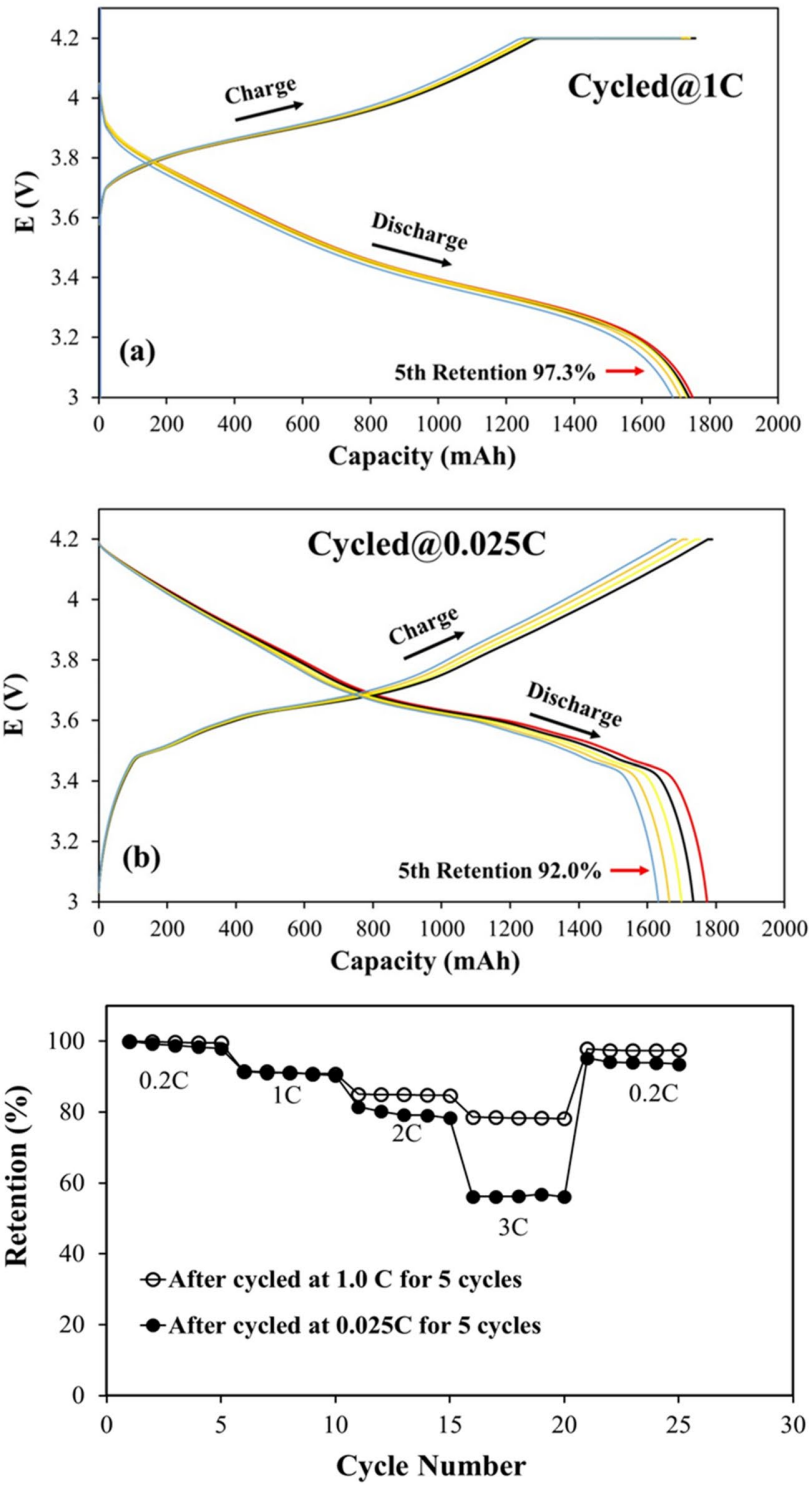
Galvanostatic charge/discharge at (**a**) 1 C and (**b**) 0.025 C for 5 cycles of the NMC532/MGP cells. (**c**) The retention of rate capability of the NMC532/MGP cells after initially cycled at 1 C and 0.025 C for 5 cycles, respectively.

The 18650-type cylinder NMC532/MGP cells activated formation conditions at 0.2 C were used to estimate the impact of rate capability on the electrochemical performance. The voltage-capacity curves fort the cells cycled 5 times at 1.0 C (named 1.0 C cell) and 0.025 C (named 0.025 C cell) after formation are shown in Fig. 1a,b, respectively. The result indicates that the cells start the charging and discharging process from 3.6 V and 4.1 V for 1 C rate, and start from 3.0 V and 4.18 V in the case of 0.025 C rate for all 5 cycles, respectively. The variations in the voltage at the initial stage of charge and discharge of the cells are primarily caused by the specific current produced by the cell polarization behaviour. The discharge capacities at 0.025 C 1st cycle is a little larger than that at 1.0 C, but the capacity fading at 0.025 C (5th retention 92%) is higher than that at 1.0 C (5th retention 97.3%). Figure 1c, displays the rate capability of NMC532/MGP cells fabricated with PP/PE/PP separator for 0.025 C cell and 1.0 C cell, respectively. Subsequently, the batteries are charged and discharged at current densities with the ranging from 0.2 to 3 C (0.2 C, 1.0 C, 2.0 C, 3.0 C) and return to 0.2 C for 5 cycles each step. Similar to previous studies^18–20^, the reduced capacity at a high current rate with increasing cycle number, is due to the increased polarization behaviors resulting from the thicker solid electrolyte interlayer (SEI) and lower Li-ion diffusion rate between cathode and anode, etc. In Fig. 1c, the Coulombic efficiency of the cell cycled at a high rate (1 C) achieve 100% retention of the unit-value stability, with increasing the cycle number and attributing higher discharge capacity to reduce polarization behaviour. To compare with Fig. 1c, the curve of 0.025 C cycled cell tends to have a lower discharge capacity but a similar discharge characteristic at 0.2 C, 1 C and 2 C. However, significant deterioration was observed in the discharge capacity of 3 C and a drop-in capacity for 5 C in Fig. 1c, the curve for the cell cycled at a low rate (0.025 C). The NMC532/MGP cell cycled at 1 C demonstrated a higher initial discharge capacity and Coulombic efficiency with a lower decay rate in comparison to the 0.025 C cycled cell. When the C-rate returned to 0.2 C, the capacities of both the cells were restored, suggesting that the cell remained more stable after 1.0 C, than the cell after 0.025 C, both are 5 cycled.

**Figure 2:**
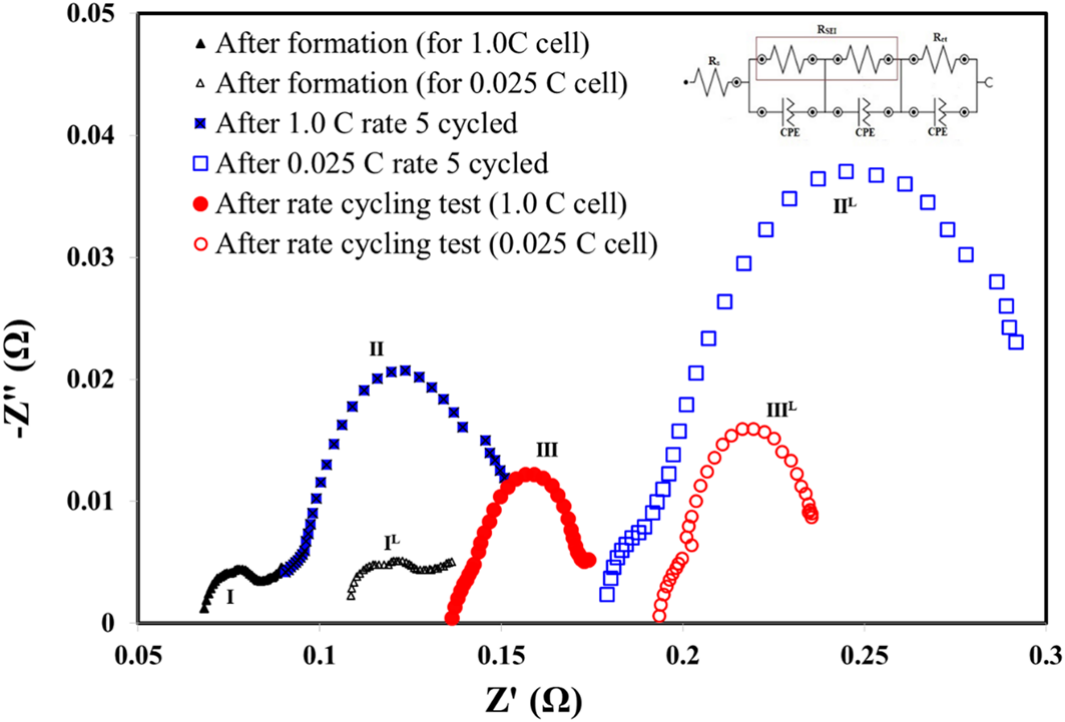
EIS plot of 1.0 C cell and 0.025 C cell after different cycled history. Inset shows the equivalent electrical circuit.

Figure 2 shows the full cell Nyquist plot of the 1.0 C cell and 0.025 C cell after three cycled stage, (I) formation cycle, (II) after the cells cycled for 5 times and (III) after the rate performance test, respectively. For the cycled stage I, II, and III, the results of 0.025 C cell are denoted as "L" superscript. The curve in the high-frequency region reflects the equivalent serial resistance (R_s_) at the electrode, electrolyte, separator interface, and contact resistance i.e. the bulk (internal) resistance. In the middle frequency region, the semicircle diameter indicates the charge transfer resistance (R_ct_) and the solid electrolyte interphase resistance (R_SEI_) resulting from the electrochemical reaction at the electrode and electrolyte interface. The inset in Fig. 2 shows the equivalent circuit to further analyse the EIS spectrum. SEI film capacitance and charge transfer are represented by the constant phase elements (CPE) in the circuit. The fitted R_s_, R_ct_ and R_SEI_ from the equivalent circuit are summarised in Table 1. The R_s_ of 0.025 C cell and 1.0 C cell after formation was 0.108 Ω and 0.068 Ω, respectively. The difference is mainly due to contact resistance by the cell manufacture processes such as electrode welding and separator assembling. The semicircle curves for 0.025 C cell and 1.0 cell shift toward the right from stage I to stage II. After the rate performance test (stage III) the semicircle curves shift toward right even more. This is due to the increase of the R_s_ of the cells which is common as the cycling number increases. Comparing the increase of R_s_ from stage I to stage II, the 0.025 C cell shows a larger increase than the 1.0 C cell. In other words, at low charging/discharging current density the resistance from the electrode, electrolyte, separator interface, and contact resistance increases more. The result could probably be attributed to the change of the separator interface or the property of separators such as the swelling or structural change in the polymer separator. The SEM analysis discussed in the following section support this hypothesis. The R_SEI_ of the 0.025 C cell was reduced after the rate cycling performance test as shown in Table 1. The R_SEI_ value of both cells on the anode (R_anode_) is higher than on cathode (R_cathode_), indicating the higher possibility of electrolyte decomposition on the anode surface after extensive cycling resulting from the formation of SEI onto the anode electrode materials.

**Table 1:** The resistance values estimated from the equivalent circuits of EIS curves.

Cycling (C/DC) conditions	R_s_ (mΩ)	R_SEI_		R_ct_ (mΩ)
		R_Cathode_ (mΩ)	R_anode_ (mΩ)	
1 C				
After formation (I)	68.30	8.64	3.61	23.90
After 5 cycles (II)	90.62	6.40	18.00	48.40
After rate test (III)	136.34	3.90	27.60	24.70
0.025 C				
After formation (I^L^)	108.00	5.02	12.80	21.40
After 5 cycles (II^L^)	179.16	10.30	97.20	16.50
After rate test (III^L^)	193.10	3.07	33.80	18.00

### SEM morphology of separator

**Figure 3:**
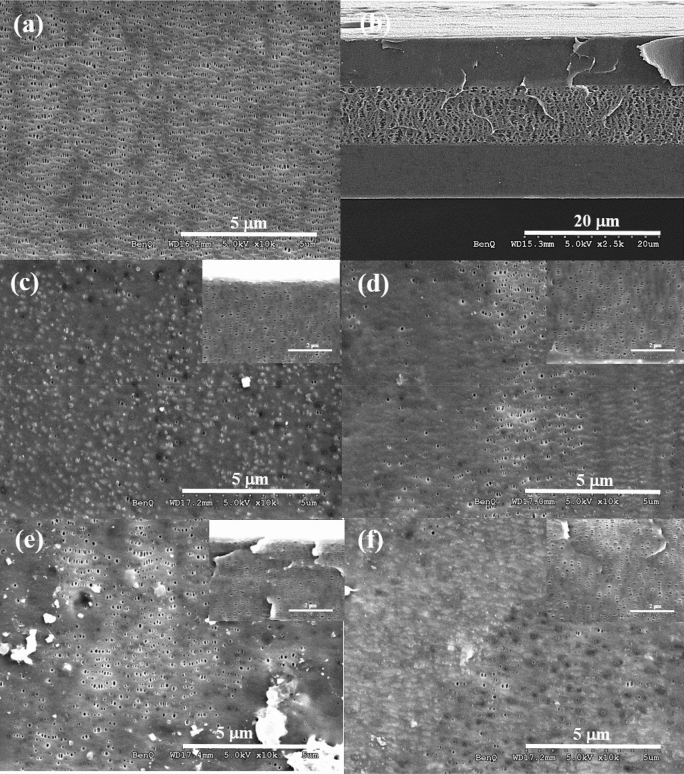
SEM images of trilayered PP/PE/PP separator: (**a**) pristine top surface; (**b**) pristine cross-section; (**c**, **e**) the cathode side image after the rate rest at 1.0 C and 0.025 C for 5 cycles, respectively; (**d**, **f**) the anode side image after the rate rest at 1.0 C and 0.025 C for 5 cycles anode side, respectively. Inset in (**c**–**f**) shows the corresponding cross-section images.

Figure 3 shows the morphology of the fresh (PP/PE/PP) separator and the separator of the 0.025 C cell and 1.0 C cell after the II cycled stage observed by high-resolution scanning electron microscope (HR-SEM). The top surface of pure separator clearly illustrates the slit pores as shown in Fig. 3a,b. Figure 3c,e represent the image of the cathode side separator surface, and Fig. 3d,f show the image of anode side separator surface. Inset images display the corresponding cross-sectional images of PP layer. Figure 3c,d are the images taken from 1.0 C cell and Fig. 3e,f are taken from 0.025 C cell. During cycling, the solid electrolyte interphase (SEI)^21^, active particles, reaction yields^11^, or lithium deposition^22^ blocks the separator pores resulting in the shrinkage of pore size. Thus, the SEM images show that the membrane surface accumulated with the rapid development of the interphase layer. Besides, the pore density from the cathode side is visibly higher than the anode side, probably due to mostly the formation of interphase on the anode surface. More importantly, the interphase layer formation on both the cathode and anode surface of 1.0 C cell is less than that of 0.025 C cell as shown in the Fig. 3c,d and in Fig. 3e,f, respectively. Fig. S1, showing the cross-section images, reveals that the pore shrinkage occurs significantly in the PP layers and negligible in the middle PE layer for both 1.0 C cell and 0.025 C cell. The SEM images (Fig. 3c–f and Fig. S1) reflect that the membranes undergo mechanical creep depending on SOC, temperature, time (number of cycles) and pressure, which leads to a strong increase in impedance and ultimately shorten cycle life and induced early field failure. This might explain partially better EIS performance of 1.0 C cell than 0.025 C cell discussed in the previous section.

We further analysed the morphology of the pores of PP/PE/PP separator in the soft-packed cell, made with lithium metal as cathode and SnTiO_2_ as the anode, cycled under charge/discharge current densities of 0.75 mA cm^−2^ g^−1^ and 1.27 mA cm^−2^ g^−1^. The cell was made to have the smooth fitting results of peak position and intensity from XRD pattern obtained by In-situ synchrotron-based high-energy x-ray diffraction experiments. The top image of insets in Fig. S2 (a) shows PP separator has lengthened (slit) pores oriented in the same direction. The elliptical pores were created by uniaxial stretching^8^ in the pure PP separator. Pure PE membrane has a uniformly interconnected highly porous structure, shown in the bottom of insets in Fig. S2a. A uniform and dense trilayered membrane can prevent dendrites growth. Fig. S2 (b) and (c) show a strong reduction in the length of the pores and partial clogging in the trilayered (PP/PE/PP) separator after charging at current densities of 0.75 mA cm^−2^ g^−1^ and 1.27 mA cm^−2^ g^−1^, respectively, as compared with pure PP and PE separator. The SEM images showing in Fig. S2 has similar results as those observed in the 18650-type cylinder NMC532/MGP cells indicating that there is also the interphase layer formation on the polymer membrane surface. The layer might come from the SEI layer composites or the electrolyte decomposition process.

### In-situ XRD analysis of separator

**Figure 4:**
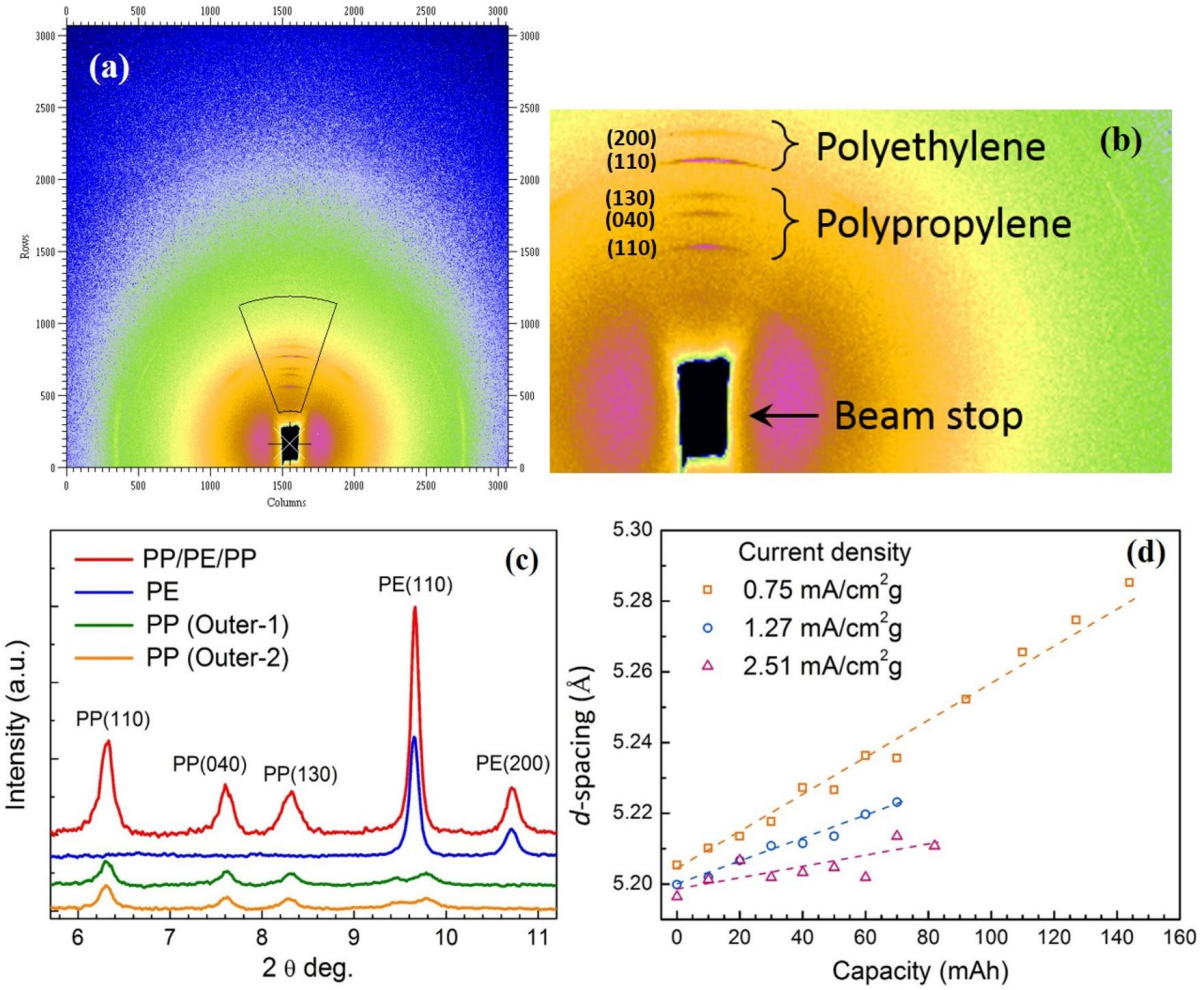
(**a**,**b**) Two dimensional (2D) XRD image of LIBs, (**c**) XRD of trilayer separator (PP/PE/PP) and its monolayer (PP and PE) and (**d**) the evolution of PP (040) on *d*-spacing respect to capacity with three different current density of 2.51, 1.27 and 0.75 mA cm^−2^ g^−1^. Data points were linear fitted as the dash lines.

The typical 2D XRD pattern of PP/PE/PP trilayer separator, (Celgard co.,) used in lithium-ion battery is shown in Fig. 4a,b, exhibiting an asymmetric image. The two main components in the separator membrane, PP and PE, contribute their characteristic diffraction peaks in the specific angles and direction as indicated in Fig. 4b. The X-ray diffraction profiles of the entire trilayered separator (PP/PE/PP) consists of its two outer monolayers (PP) and a single inner layer (PE), is revealed in the Fig. 4c. In addition to three major diffraction peaks at 2*θ* = 6.31°, 7.62° and 8.3° from the crystalline phase of PP layers which corresponding to (110), (040) and (130) plane, respectively^23^, there were also two minor peaks, at 2*θ* = 9.45° and 9.77°, embedded in the PE (110) main peak for this PP/PE/PP trilayered membrane. The other two sharp distinct peaks at around 2*θ* = 9.65° and 10.67° which corresponding to (110) and (200) plane, respectively, are indications of the crystalline structure of PE layer. The data collection of in-situ XRD is synchronized with the charging process identified by different current densities of 0.75, 1.27 and 2.51 mA cm^−2^ g^−1^. Fig. S3 illustrates the plot of cell voltage versus capacity during charging. All the three curves manifest similar charging trend, with variation in the capacity influenced by the applied current density. The electrode exhibit the reversible capacity of 220 mAh g^−1^ at a low current density of 0.75 mA cm^−2^ g^−1^, and the battery charging capacity decreases with increasing current density.

**Figure 5:**
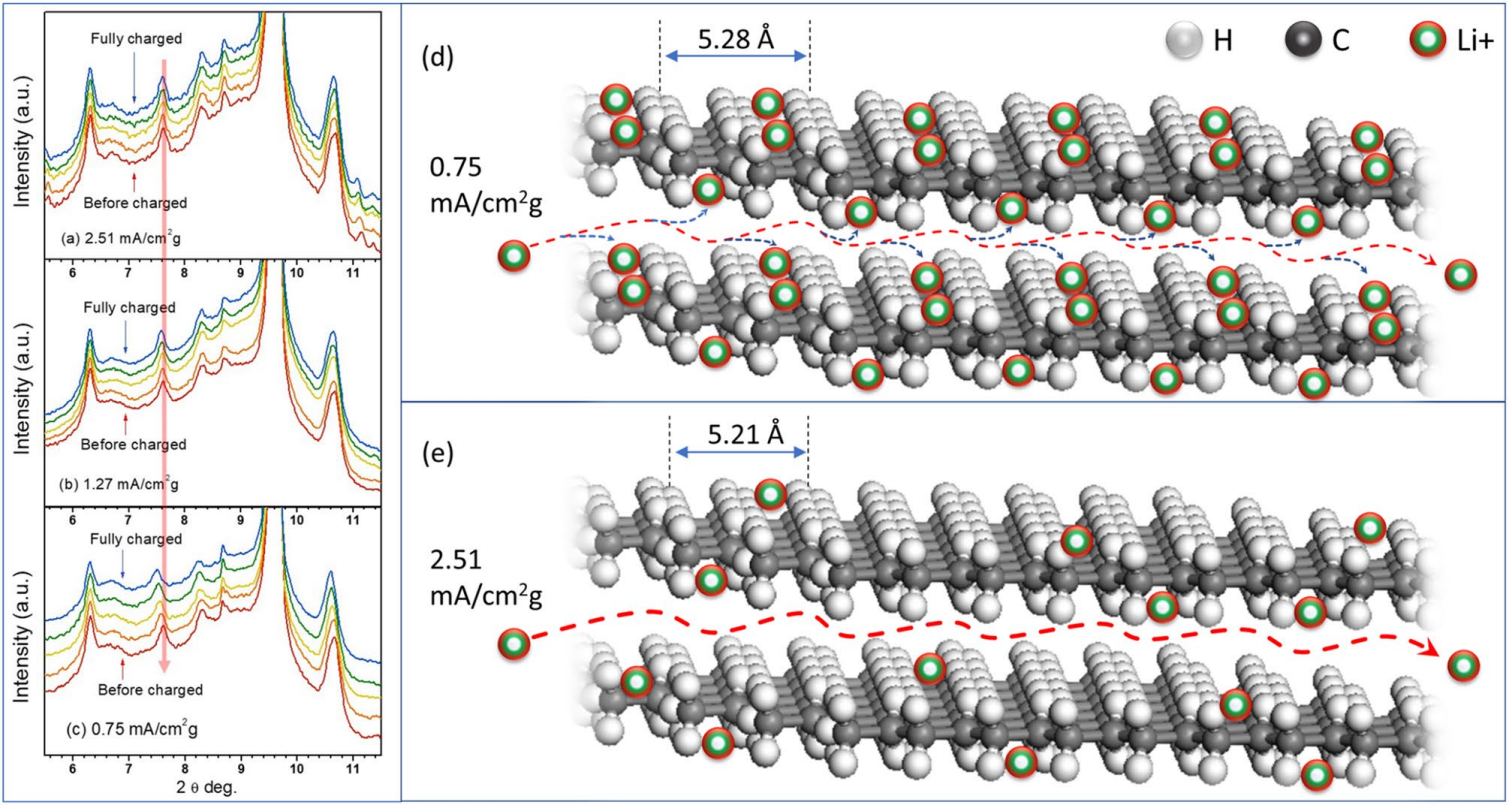
In-situ XRD at different charged states under different charging current density (**a**) 2.51, (**b**) 1.27 and (**c**) 0.75 mA cm^−2^ g^−1^. The frames are vertically shifted for clarity. The schematic representation for the structure evolution of (040) facets in the PP crystal at the charge rate of (**d**) 0.75 mA/cm^2^ g and (**e**) 2.51 mA/cm^2^ g.

Figure 5a–c shows the in-situ XRD of LIBs cycled at charging current density of 2.51, 1.27 and 0.75 mA cm^−2^ g^−1^, respectively. Highly orientated XRD patterns indicate high crystalline PP and PE structure in the PP/PE/PP trilayer membrane despite porous structure observed in SEM images. Five in-situ XRD data from equally spaced states of charge (SOC) ranging from 0 to 100% were chosen to illustrate the entire charging process for the three different charging current densities. The descending arrow in Fig. 5 indicates the same position of 2*θ* degree. The diffraction peaks of crystalline PP and PE phase are slightly shifted towards low angle with different shifting degree (2*θ*), implying that the lattice spacing enlargement is selective. For instance, the second diffraction peak of PP, which corresponding to (040) plane shifted from 2θ = 7.62° to 7.54° after fully charged, shows the largest spacing enlargement than others. Moreover, the enlargement of lattice spacing is found to be inversely proportional to the charging current density, i.e. the lower charging current density causes much significant peak shifting than the higher one. As seen in Fig. 4d, in the case of PP (040) peak the major lattice spacing enlargement is found for lower charging current density, 0.75 mA cm^−2^ g^−1^. It is also worth to notice that the lattice spacing evolution follow a monotonic increasing and continuous until the cell is fully charged. The calculated percentage of plane-spacing difference, Δ*d* = *d*_f_ − *d*_0_, for all the observed peaks corresponding to PP and PE, at different charging current densities are listed in Table 2. It reveals that the largest plane-spacing expansion up to 1.535% is originated for PP (040) peak at lowest charging current density of 0.75 mA cm^−2^ g^−1^, while PE (110) has only 0.042% plane-spacing difference at highest charging current density. In addition to peak shift, no major changes observed in the diffraction peaks for all the given current densities during the entire charging process.

**Table 2:** The *d-*spacing difference (%) for different planes at difference C-rate.

Current density (mA cm^−2^ g^−1^)	*Δd/d*_*0*_ (%)				
	PP(110)	PP(040)	PP(130)	PE(110)	PE(200)
0.75	0.127	1.535	0.764	0.198	0.527
1.27	0.063	0.448	0.253	0.083	0.234
2.51	0.048	0.276	0.006	0.042	0.103

*Δd* = *d*_*f*_ −* d*_*0*_, spacing difference.

*d*_*f*_, the spacing at fully charged condition.

*d*_*0*_, the spacing before charging condition.

The synchrotron in-situ XRD patterns shown in Fig. 5 reveal the lattice expansion (crystalline structure variation) of the trilayered separator. This denotes the corresponding plane-spacing is enlarged during the charging process. An ideal separator, however, allows only the free transport of lithium-ion and the electrolyte molecule without interacting with them. The peak shift resulted from the enlargement of crystalline lattice space indicates there is something embedded into polypropylene (PP). Besides, it should be emphasized that not all the PP peaks shifted during charging. The changes in the crystalline structure could only occur in some specific lattice plane, such as PP (040). As for polyethylene (PE) middle layer, both the main peaks of 2θ = 9.65° and 10.67° shift to 9.58° and 10.61°, respectively, when it is fully charged. These unexpected peaks shift caused by crystalline structure change of separator during the charging process, which implies that the separator used in the present case may have some distresses. From our results, we found that the crystalline structure of PP and PE in the trilayered membrane also participates in the reactions with Li-ions and electrolyte, which were formerly thought to have no function in LIB. To demonstrate the aforementioned Li^+^ intercalation upon LIB charging, for the structural evolution of (040) facets in the PP crystal at the charge rate of 0.75 mA/cm^2^ g and 2.51 mA/cm^2^ g are respectively shown in Fig. 5d,e.

From the in-situ XRD data, it was known that an unexpected current density-dependent plane-spacing enlargement found during the battery charging process. We, therefore, proposed a rational mechanism to explain the reason for the expansion of crystalline lattice constant during charging, the selective enlargement effect on the specific crystalline planes (some peaks are more evident) and the current density-dependent lattice expansion. Previous XRD (in-situ XRD) studies^24–26^ on electrode structural change revealed that the lattice expansion was found during charging/discharging through the interaction and intercalation/deintercalation of lithium ions in electrode materials. From our results, we proposed a similar phenomenon on ions induced lattice spacing expansion can also be found in the separator. Considering the lithium-ion transport in LIBs, cathode and anode act as terminals that release and collect the lithium ions, respectively, while the separator in between directs and regulates the transport of lithium ions through its porous structure. Moreover, the pores can significantly shrink and eventually enclose under stress resulted from the compress of electrodes, leading to a reduction in the effect of the lithium-ion pathway. Not only these pores provide a pass way for ions transportation, but the amorphous and crystalline portions of these separators can also be the choice for Li-ion movement.

### DFT calculations

From the small-angle in-situ XRD pattern for the trilayer membrane, three main distinguishing features were found. The first is the peak shifted toward low angle for both crystalline part of PP and PE in the separator, the second is that the peak shifting of PP is larger than that of PE and the last is that the PP(040) peak has the maximum peak shift. From the DFT calculation, the mechanical properties of PP and PE were first calculated to understand the anisotropic elastic behaviour, it could be one of the reasons that responsible for the extent of peak shifting of PP and PE. Experiments suggest that the peak shifting is due to the insertion of Li ions into the crystalline lattice of PP and PE. To prove this hypothesis, reaction energy after the Li-ion insertion or intercalation was calculated by DFT method. In this study, finite temperature effect was not included in the calculations. The negative reaction energy indicated that the insertion/intercalation is thermodynamically favourable and hence supports the suggestion.

**Figure 6:**
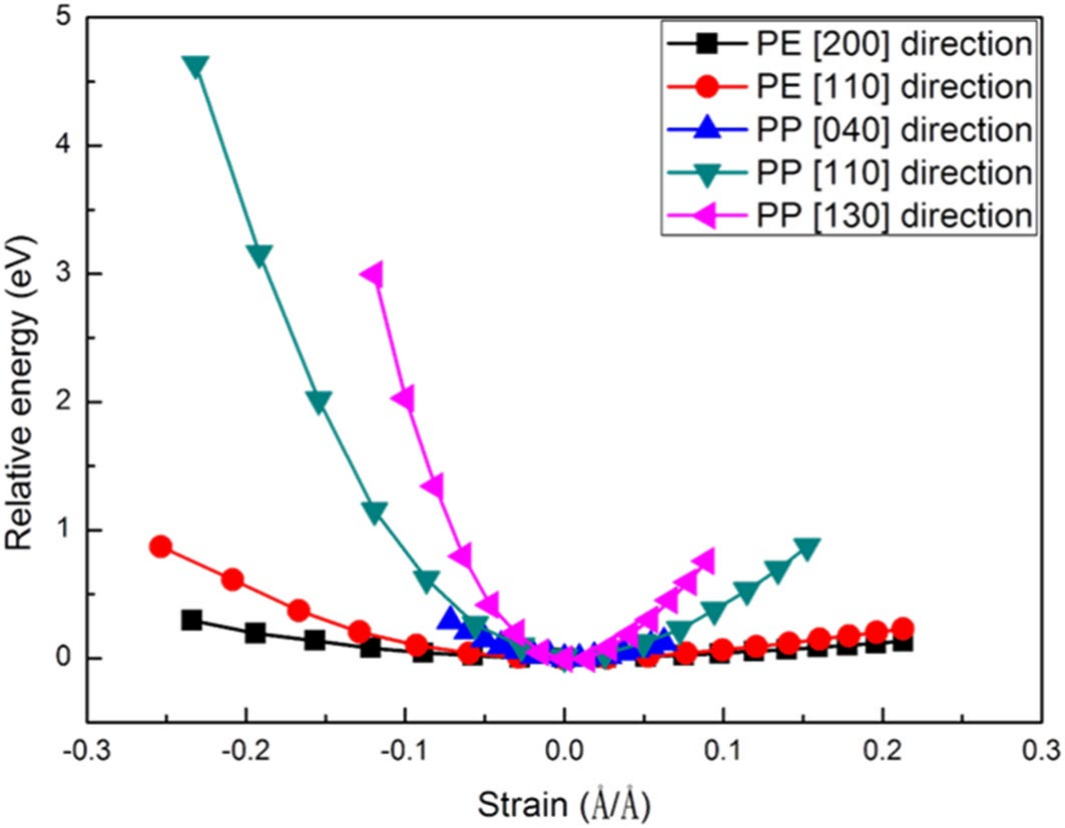
Relative energy-strain curves for PE in [200] and [110] direction and for PP in [040], [110] and [130] direction.

From the experiments, it was observed that the peak shifting toward small angle is larger in PP than that of PE indicating that larger lattice expansion in the outer PP layer than in PE after charging. The XRD pattern also shows that each diffraction lattice plane has a different extent of peak shifting, specifically PP (040) showing the major peak shifting. Hence, the energy-strain curve of PE and PP was calculated by considering compressing and stretching the lattice in the [110] and [200] directions for PE and [110], [040] and [130] directions for PP, respectively. Here, compressing and stretching denotes the negative and positive strain values, respectively, in Fig. 6. The directions were chosen based on the diffraction patterns observed in Fig. 4c. The proposed schemes for the lattice expansion/contraction of PE and PP are illustrated in Figs. S4 and S5, respectively. The strain-dependent relative energy curves for different direction are plotted in Fig. 6 for both PE and PP. The relative energy showing in the y-axis is the energy which set zero-strain energy as zero. Figure 6 shows that the curvature of relative energy-strain in PE for both [200] and [110] are smaller than that of strain in PP for [040], [110] and [130]. The results indicate that strain caused is easier in PE than in PP, since small curvature represents small elastic constant. If comparing the curvatures of individual polymer, the strain caused along [200] in PE is easier than along [110] and strain triggered along [040] in PP is easier than along [110] and [130].

Comparing calculated (simulated) data with experimental data shown in Table 2, some results are consistent between theoretical calculation and experiments, but with few exceptions. The consistent part is that for the individual polymer the plane with the highest peak shifting its normal vector also has the lowest curvature of the relative energy-strain curve. For example, PE (200) shows 0.527% *d-*spacing difference which is larger than 0.198% of PE (110) and the curvature of PE in [200] is also smaller than that of PE in [110]. In the case of PP, the plane PP (040) shows the highest *d-*spacing difference of 1.535% and the curvature of PP in [040] is also the smallest among all the three directions. The inconsistent part from Fig. 6, is that in theoretical calculation all the curvatures in PE are smaller than PP, but experiment data in Fig. 5 and Table 2, shows that PP has larger *d-*spacing difference than that of PE. For example, PP (044) shows higher peak shifting than PE (200). Thus, anisotropic mechanical properties could only explain the *d-*spacing difference in various planes of individual polymer, but not the overall behavior of the trilayer separator. There should be another factor, such as insertion of Li-ions or other species into crystalline part of PP or PE, that responsible for the peak shifting phenomena observed in the in-situ XRD experiments.

To investigate whether the lattice expansion is due to the insertion effect, possible intercalation species such as Li^+^, EC, F^−^, LiF, PF_5_ from electrolyte were considered. To confirm whether the insertion is thermodynamically favourable, the total energy difference (reaction energy) before and after insertion calculated by DFT method, was used as an index to revealing the tendency. Thus, the following reaction equations were considered to study insertion effects in PE.

1 $${\text{PE}} + {\text{LiPF}}_{{6}} \to ({\text{PE-Li}}^{ + } ) + {\text{PF}}_{{6}}^{ - }$$

2 $${\text{PE}} + \left( {{\text{4EC-Li}}^{ + } } \right) \to \left( {{\text{PE-Li}}^{ + } } \right) + {\text{4EC}}$$

3 $${\text{PE}} + {2}\left( {{\text{4EC-Li}}^{ + } } \right) \to \left( {{\text{PE-2Li}}^{ + } } \right) + {\text{8EC}}$$

4 $${\text{PE}} + {3}\left( {{\text{4EC-Li}}^{ + } } \right) \to \left( {{\text{PE-3Li}}^{ + } } \right) + {\text{12EC}}$$

5 $${\text{PE}} + \left( {{\text{4EC-Li}}^{ + } } \right) \to \left( {{\text{PE-EC}}} \right) + \left( {{\text{3EC-Li}}^{ + } } \right)$$

6 $${\text{PE}} + \left( {{\text{4EC-Li}}^{ + } } \right) \to \left( {{\text{PE-EC-Li}}^{ + } } \right) + {\text{3EC}}$$

7 $${\text{PE}} + {2}\left( {{\text{4EC-Li}}^{ + } } \right) \to \left( {{\text{PE-EC-2Li}}^{ + } } \right) + {\text{7EC}}$$

8 $${\text{PE}} + \left( {{\text{4EC-Li}}^{ + } } \right) \to \left( {{\text{PE-2EC-Li}}^{ + } } \right) + {\text{2EC}}$$

9 $${\text{PE}} + {2}\left( {{\text{4EC-Li}}^{ + } } \right) \to \left( {{\text{PE-2EC-2Li}}^{ + } } \right) + {\text{6EC}}$$

10 $${\text{PE}} + {\text{PF}}_{{6}}^{ - } \to ({\text{PE-F}}^{ - } ) + {\text{PF}}_{{5}}$$

11 $${\text{PE}} + {\text{LiPF}}_{{6}} \to ({\text{PE-LiF}}) + {\text{PF}}_{{5}}$$

12 $${\text{PE}} + {\text{LiPF}}_{{6}} \to ({\text{PE-PF}}_{{5}} ) + {\text{LiF}}$$

Equation (1) describes the possibility of Li-ion insertion into PE crystal with the lithium salt, LiPF_6_, as the lithium source. Equation (2)–(4) also describe the insertion of Li-ion into PE crystal but with four EC solvated Li-ion, as the lithium source. Isolated Li-ion is rare to be found in the electrolyte due to its high tendency to solvate with EC thus it was not considered in this study. From Eqs. (2) to (4) the insertion concentration of Li ions increases from 1.39 to 2.78 and 4.17 per 100 PE monomers, respectively. Equation (5) describe the insertion of EC with one of the four EC molecules that solvate Li-ion as the EC source. Normally the electrolyte composed of EC and other linear molecules with low viscosity. The size of those linear molecules are too big to insert into the PE crystal lattice, thus the only insertion of EC was considered. Equations (6) to (9) describes the co-insertion of Li-ion and EC into PE crystal. Since if either Li-ion or EC molecule can insert into PE crystal, there will be a higher possibility of co-insertion also to occur. From Eqs. (6) to (9), the concentrations of inserted Li-ion and EC are 1.39 and 1.39, 1.39 and 2.78, 2.78 and 1.39, and 2.78 and 2.78 per 100 PE monomers, respectively. The lithium salt is one of the major species in electrolyte, hence considering the possibility of insertion of lithium salt or its derived species is necessary to completely check all the possible insertion cases. Equation (10) explain the insertion of F-ion with PF_6_^−^ as the F-ion source. The lithium salt, LiPF_6_, was chosen to match with the experimental conditions. Since the size of LiPF_6_ is too large, the insertion of LiPF_6_ is highly unfavorable. However, the LiPF_6_ usually dissociate to Li-ion and PF_6_^−^ in the electrolyte, thus PF_6_^−^ was considered as the F-ion source. LiF and PF_5_ were found in the electrolyte are small enough to insert into PE crystal. Thus, Eq. (11) consider the insertion of LiF molecule with LiPF_6_ as the LiF source and Eq. (12) consider the insertion of PF_5_ molecule with LiPF_6_ as the PF_5_ source.

After the above listed possible insertion reactions, the models of each reactant and product was built to calculate the total energy of each model. The reaction energy was obtained by simply subtracting the sum of the total energy of products with the sum of the total energy of reactants. The model shown in Fig. S6 (a) was taken from Eq. (2) as an example, to illustrate the calculation scheme. First one in the reactant side, we proposed the supercell model to show the crystalline PE unit cell. The grey colour boxes indicate the boundaries of the three dimensional PE crystal model, which is repeated periodically. The second from left shows the 4EC-Li^+^ model, representing the lithium-ion solvated by four EC molecules. The first one in the product side, shows the Li-ion intercalated crystalline PE model. Several insertion sites were tested according to the symmetry to ensure the lowest energy model was obtained. The second one (model) in the product side shows the EC molecule. Though the four EC molecules were displayed together, the energy was obtained by calculating one EC molecules in implicit solvent and then multiplied by four. Fig. S6 (b) illustrate the models of ions, molecules and clusters used in the reaction energy calculations. The detailed DFT methods to calculate the total energy of each model was described in “DFT calculations” section.

Table 3 shows the calculated reaction energy on insertion effects of PE. Considering Li-ion as insertion species, it is easier to insert Li-ion into the PE crystal if it comes from EC solvated Li-ion, and not from the lithium salt. The possibility of the highest concentration of Li-ion insertion is 2.78 ions per 100 PE monomers. In the case of EC insertion, since the obtained reaction energy is positive it is hard to insert EC into PE crystal. In the case of co-insertion, the reaction energies are negative in all the tested conditions, which is indicating that co-insertion have high possibility to occur. The insertion of F ion, LiF or PF_5_ into PE crystal are impossible due to the obtained very high reaction energies. Among all the cases tested in these calculations, the co-insertion of Li-ion and EC molecule respectively with 1.39 ion and 1.39 molecule per 100 PE monomers, has the most negative reaction energy. This insertion model was thus chosen to perform a simulated XRD analysis to compare with the pattern obtained from experimental XRD.

**Table 3:** Reaction energies of Li-insertion effects in PE.

Insertion species	Reactant		Product		Intercalation density of EC (EC/100 PE monomers)	Intercalation density of Li (Li/100 PE monomers)	Reaction energy (eV)
Li ion	PE	LiPF_6_	PE-Li^+^	PF_6_^−^	–	1.39	0.41
	PE	(4EC-Li^+^)	PE-Li^+^	4EC	–	1.39	− 0.78
	PE	2(4EC-Li^+^)	PE-2Li^+^	8EC	–	2.78	− 0.62
	PE	3(4EC-Li^+^)	PE-3Li^+^	12EC	–	4.17	0.7
EC	PE	(4EC-Li^+^)	PE-EC	3EC-Li^+^	1.39	–	0.28
Li ion	PE	(4EC-Li^+^)	PE-EC-Li^+^	3EC	1.39	1.39	− 1.22
+	PE	2(4EC-Li^+^)	PE-EC-2Li^+^	7EC	1.39	2.78	− 1.08
EC	PE	(4EC-Li^+^)	PE-2EC-Li^+^	2EC	2.78	1.39	− 0.19
	PE	2(4EC-Li^+^)	PE-2EC-2Li^+^	6EC	2.78	2.78	− 0.88
others	PE	PF_6_^−^	PE-F^−^	PF_5_	–	–	8.16
	PE	LiPF_6_	PE-LiF	PF_5_	–	–	4.36
	PE	LiPF_6_	PE-PF_5_	LiF	–	–	2.82

**Figure 7:**
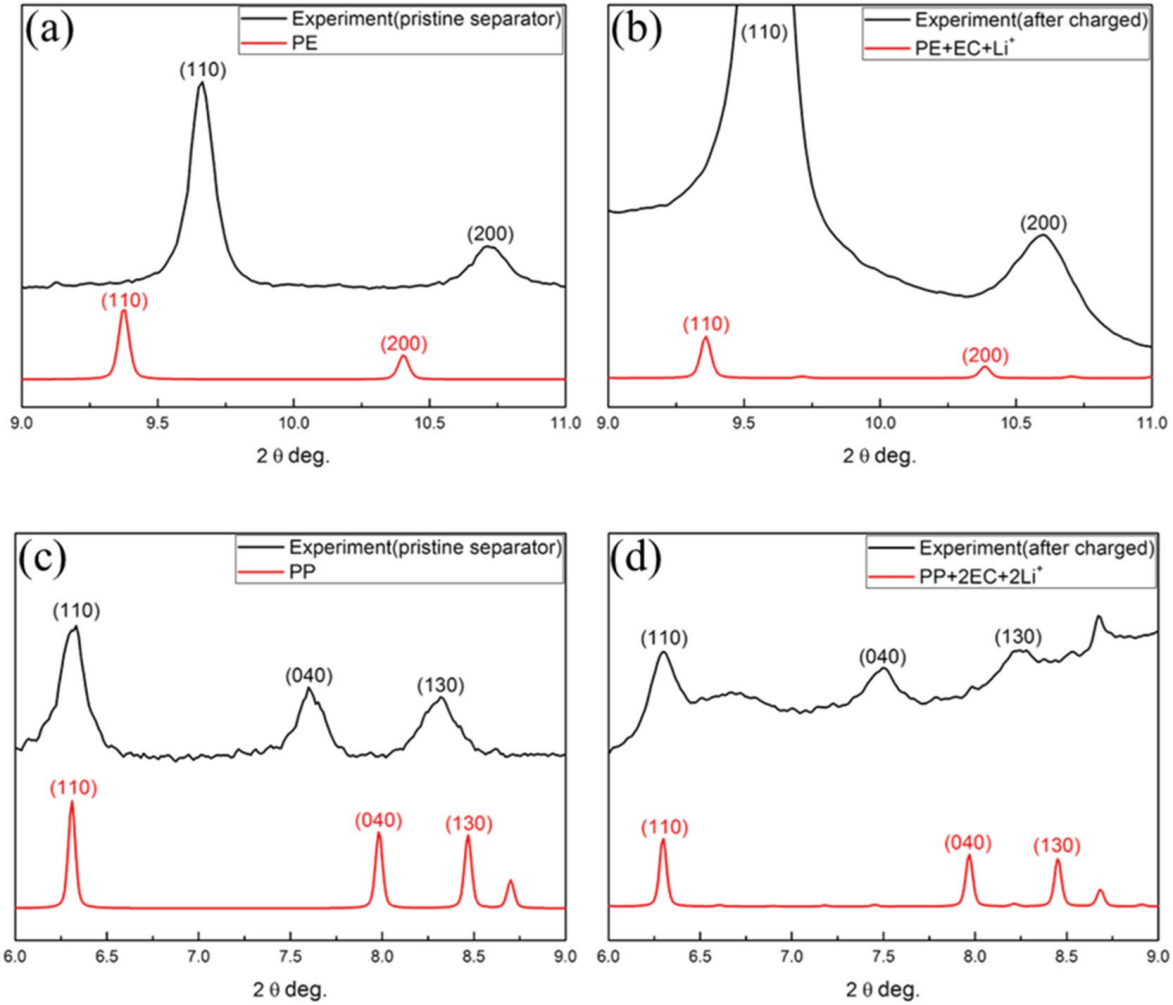
Comparison of simulated XRD pattern of (**a**) pure PE, (**b**) PE-EC-Li^+^ model, (**c**) pure PP and (**d**) PP-2EC-2Li^+^ model.

Figure 7a shows the diffraction pattern of PE from the pristine separator and simulated diffraction pattern of pristine PE from DFT calculation. The *d*-spacing difference between DFT calculation and experiment peak are 3.301% and 2.991% for PE (110) and PE (200), respectively. DFT calculation slightly overestimates the lattice parameters, which is common in GGA method. Figure 7b shows the diffraction pattern of PE after fully charged and simulated diffraction pattern of PE-EC-Li^+^ model from DFT calculation. In this case, the *d*-spacing difference between DFT calculation and experiment peak are 2.442% and 1.991% for PE (110) and PE (200), respectively. The amount of lattice expansion due to co-insertion is around 0.176%, and was listed in Table 4. Since cubic cell was maintained during relaxation for the good convergence of the charged system, the lattice expansion in x, y and z directions was the same in the calculation. To comparison of this result with Table 2, the trend is qualitatively consistent with the experiments. Here, the DFT calculations were assumed a smaller lattice expansion for PE, while Li-ion and EC insertion takes place. This might be due to the fact that, the DFT calculation originally implicit a larger lattice parameter for pristine PE than the experiment and thus expected availability of more interatomic space in the DFT calculated PE lattice. This might give more space for the Li insertion in the PE lattice. Considering these insertion models, DFT would expect a smaller lattice expansion than an experiment. The other distinct feature of simulated XRD pattern of insertion model is the perturbed peaks at 2θ = 9.7° and 10.7°. These perturbed peaks are due to local rearrangement near the insertion sites. Figure 7b also shows a tiny hump near PE (110) and PE (200) peaks. Thus, the DFT calculation models proposed here confirms the insertion of Li-ion and EC into the crystalline lattice of PE.

**Table 4:** Difference in the lattice constant of PE.

	a (Å)	b (Å)	c (Å)	△a (%)	△b (%)	△c (%)
PE	7.607	5.071	2.536	–	–	–
PE-EC-Li^+^	7.620	5.080	2.540	0.176	0.176	0.176

In addition, the insertion effect of PP was studied by the same scheme as that of PE and thus 12 identical reactions were considered, but replacing PE with PP and similar calculation scheme was also performed. Table 5 shows the reaction energy of insertion effects of PP. From the results, it was indicated that Li-ion insertion is feasible for the ion motility from EC solvated Li-ion. The highest concentration of Li-ion insertion is 1.85 ions per 100 PP monomers. Regardless, EC insertion is not possible due to the obtained positive reaction energy, therefore the correlation of co-insertion of Li-ion and EC is also conceivable. The most probable case is co-insertion of Li-ion and EC with 1.85 ions and 1.85 EC molecules per 100 PP monomer. Apart from that, the insertion of F ion, LiF or PF_5_ into PE crystal are found to be impossible due to very high reaction energies. Thus the most possible insertion model was chosen to perform simulated XRD analysis to compare with the experimental XRD pattern.

**Table 5:** Reaction energies of Li-ion insertion effects in PP.

Insertion species	Reactant		Product		Intercalation density of EC (EC/100 PP monomers)	Intercalation density of Li (Li/100 PP monomers)	Reaction energy (eV)
Li ion	PP	LiPF_6_	PP-Li^+^	PF_6_^−^	–	0.93	0.25
	PP	(4EC-Li^+^)	PP-Li^+^	4EC	–	0.93	− 0.93
	PP	2(4EC-Li^+^)	PP-2Li^+^	8EC	–	1.85	− 1.2
	PP	3(4EC-Li^+^)	PP-3Li^+^	12EC	–	2.78	− 0.58
EC	PP	(4EC-Li^+^)	PP-EC	3EC-Li^+^	0.93	–	0.27
Li ion	PP	(4EC-Li^+^)	PP-EC-Li^+^	3EC	0.93	0.93	− 1.15
+	PP	2(4EC-Li^+^)	PP-EC-2Li^+^	7EC	0.93	1.85	− 1.4
EC	PP	3(4EC-Li^+^)	PP-EC-3Li^+^	11EC	0.93	2.78	− 0.37
	PP	(4EC-Li^+^)	PP-2EC-Li^+^	2EC	1.85	0.93	− 0.92
	PP	2(4EC-Li^+^)	PP-2EC-2Li^+^	6EC	1.85	1.85	− 1.82
	PP	3(4EC-Li^+^)	PP-2EC-3Li^+^	10EC	1.85	2.78	− 1.44
others	PP	PF_6_^−^	PP-F^−^	PF_5_	–	–	7.87
	PP	LiPF_6_	PP-LiF	PF_5_	–	–	4.34
	PP	LiPF_6_	PP-PF_5_	LiF	–	–	3.08

Figure 7c shows the diffraction pattern of PP from the pristine separator and simulated diffraction pattern of pristine PP from DFT calculation. Three distinct diffraction peaks obtained from the calculation are consistent with experimental peaks with slightly a low-angle shift of (110) peak and slightly high-angle shift of (040) and (130) peak. The errors obtained between calculation and experiments are 0.364%, − 4.78% and − 1.79% in (110), (040) and (130) peak, respectively. Thus, the DFT calculation in the PP case projected a small lattice parameter value along *y*-direction than the *x*-direction (shown as Fig. S5) compare to experimental observation. This might due to the complicate arrangement of CH_3_ group present. Figure 7d shows the diffraction pattern of PP after fully charged and simulated diffraction pattern of PP-2EC-2Li^+^ model obtained from the DFT calculation. The *d*-spacing difference between DFT calculation and experiment are 0.009%, − 5.842% and − 2.381% for PP (110), PP (040) and PP (130) peaks, respectively. Table 6 shows the amount of lattice expansion found to be around 0.182%. Comparing this result with Table 2, the trend is consistent with experiments qualitatively. The other distinct feature of simulated XRD pattern of insertion model is the perturbed peaks at 2θ = 6.6°, 7.3°, 7.5°, 8.2° and 8.8°. These perturbed peaks are due to local rearrangement near the insertion sites. Figure 7d also shows a small hump near to PP (110) and PP (040) peaks. Thus, our DFT calculations confirm the insertion/intercalation possibility of Li-ion and EC into the crystalline lattice of PP.

**Table 6:** Difference in the lattice constant of PP.

	a (Å)	b (Å)	c (Å)	△a (%)	△b (%)	△c (%)
PP	6.606	19.817	6.606	–	–	–
PP-2EC-2Li^+^	6.618	19.853	6.618	0.182	0.182	0.182

From the DFT calculations, the co-insertion of Li-ion and EC are thermodynamically favourable for both PE and PP polymer. The insertion/intercalation of these ions, thus, provides the driving force to expand the lattice. Besides, the lattice expansion is larger in PP than that in PE while co-insertion. This result consistent with the experimental results (SEM and XRD) showing that the outer PP layer has a larger peak shifting toward low angle than that of the PE layer. Comparing the reaction energies listed in Table 3 for PE with the reaction energies listed in Table 5 for PP, it was revealed that the reaction energy for the co-insertions of Li-ion and EC into PP is lower than that into PE. Therefore, experimentally the peak shifting in PP could be evidently detected in the in-situ XRD pattern. More importantly, the calculation results concern from the thermodynamics aspect. This explains that the lowest current density of 0.75 mA cm^−2^ g^−1^ in this study has largest peak shifting since high charge/discharge rate might not provide enough time for Li-ion and EC molecule to insert into the PP or PE lattice.

## Conclusion

In this study, the effect of very low charge/discharge current (0.025 C) on the separator of the LIB system was investigated. The EIS results show the increase in impedance and ultimately shorten cycle life at very low charge/discharge current compared to that at normal charge/discharge current (1 C). The SEM images illustrate that the interphase layer formation on both the cathode and anode surface of 1.0 C cell is less than that of 0.025 C cell. The in-situ XRD data demonstrate that an unexpected current density-dependent enlargement of plane-spacing of crystalline PP and PE occurred during the battery charging/discharging process. Specifically, the enlargement is more obviously at the very low charge/discharge current case. The result also demonstrates that the structural change of the trilayered membrane is irreversible after many cycles. The DFT calculations show that the co-insertion of EC molecular and Li^+^ is thermodynamically favourable reaction in both PP and PE. The simulations also demonstrate the lattice expansion in the co-insertion models. The simulated XRD pattern displays the shift in the peaks of PE (200) of the PE-EC-Li^+^ model and PP (040) of PP-2EC-2Li^+^ model, which are compatible with the in-situ XRD results. The calculations concern from the thermodynamics aspect. This explains that the lowest current density of 0.75 mA cm^−2^ g^−1^ (equivalent to 0.025 C) in this study has largest peak shifting, since high charge/discharge rate might not provide enough time for Li-ion and EC molecule to insert into the PP or PE lattice. The insertion of lithium ions into the separator might be one of the reasons that contribute to the degrading of the LIB performance at a very low rate. Therefore, designing the new separator material with a more compact crystalline structure or surface modification to reduce the Li insertion during the battery operation is desirable.

## Materials and methods

### Electrodes and electrolyte preparation

The 18650-type cylinder batteries were assembled with Li_1.02_Ni_0.50_Mn_0.30_Co_0.20_O_2_ (NMC-532 from Umicore, Korea) as cathode electrode and mesophase graphite (MGP, China Steel Chemical Co., Taiwan) as anode electrode with trilayer (PP/PE/PP) separator (25 μm thickness, Celgard 2325, denoted as Celgard). The cathode electrode had a density of 3.45 g cm^−3^, composed of 93.4 wt% NMC532, 1.5 wt% Super-P, 1 wt% VGCF, 4 wt% PVDF and 0.1 wt% H_2_C_2_O_4_, and coated on an aluminium foil. The negative electrode (density 1.61 g cm^−3^) made of 94.9 wt% MGP, 1 wt% SP, 4 wt% PVDF and 0.1 wt% H_2_C_2_O_4_, coated on copper foil. The thickness was 125 μm and 129 μm for cathode and anode electrodes, respectively. The electrolyte solution contains lithium salt (1 M LiPF_6_) in ethylene carbonate/dimethyl carbonate (EC/DMC 1:1 by weight). The prepared electrodes and electrolyte were stored in a glove box under argon atmosphere. The galvanostatic charge/discharge tests were measured using a battery automatic tester (Chroma ATE Inc., Taiwan) with a voltage window of 3.0–4.2 V and every cell was activated formation conditions at 0.2 C (400 mA) 3 cycles, then cycled for five C/DC cycles at low rate 0.025 C and high rate 1.0 C. Electrochemical impedance spectroscopy (EIS, Autolab PGSTAT30, Eco Chemie) analysis was done to measure the interfacial resistance between the separator and electrode, at the state of charge (SOC) 50% for three different test conditions such as, after formation, after 5 cycles at 1 C or 0.025 C and after the rate performance tests, in the frequency range 0.1 Hz to 1 MHz. A high-resolution scanning electron microscopy (SEM, HITACHI S-4300) investigated both the top and cross-section morphology of the trilayer (PP/PE/PP) organic membrane (Celgard 2325). HR-SEM was employed to analyse the fresh separator and the separator retrieved after the rate capability test of low (0.025 C) and high (1.0 C) rate 5 cycled cells.

### In-situ X-ray diffraction studies

Standard batteries can be investigated with synchrotron-based high-energy XRD without modification^27, 28^. The transmission mode measurements are performed to obtain 2D-diffraction pattern. The high-energy two-dimensional X-ray powder diffraction (2D XRD) is widely used to measure the lattice strain^26^. In this work, the low-angle in-situ X-ray diffraction was adopted to examine the change in the lattice constant of the separator during the cell charging. Here, the experiments were conducted on SP12B2, SPring8 in Japan. The soft-packed LIB is composed of lithium metal as a cathode electrode, SnTiO_2_ as anode electrode^29, 30^, and the mixed solvent of ethylene carbonate and diethyl carbonate (EC/DEC 1:1 by weight) with 1 mol dm^−3^ LiPF_6_ as an electrolyte. Here, SnTiO_2_ as the anode electrode material instead of commercial graphite was used to have a good signal-to-noise ratio so that the smooth fitting results of peak position and intensity could be obtained. At low-angle XRD, the graphite anode would cause the detection of non-smooth and noisy diffraction peaks due to the low signal-to-noise ratio of the desired signal. The soft-packed LIB is placed in an Autolab PGSTAT204 electrochemical testing holder and irradiated by the synchrotron X-ray with the wavelength of *λ* = 0.68968 Å with the theta resolution is 0.01°, and the X-ray flux is ~ 10^13^ cm^−2^ s^−1^. For both the X-ray window and current collector^31^, a conductive Kapton foil was used to isolate the humidity and air. The distance between the sample and the MAR CCD rapid scan (two-dimensional, 2D) is 260.11 mm which is maintained throughout the experiment.

### DFT calculation methods

First-principles calculation was applied in the DFT framework, which was implemented in the Vienna Ab initio Simulation Package (VASP) using a generalized gradient approximation (GGA) parameterized by Perdew-Burke and Ernzerhof (PBE)^32–35^. The projector augmented wave (PAW) potentials^36, 37^ were employed to define the atomic centre potential. The criterion for geometry optimization was defined as the action of the Hellman–Feynman forces on atoms that is less than 0.001 eV/Å. The size of crystalline PE model used in this analysis was 15.20 Å × 15.20 Å × 15.20 Å as composed of 2 × 3 × 6 unit cells with a total of 432 atoms and 19.84 Å × 19.84 Å × 19.84 Å as composed of 3 × 1 × 3 unit cells with a total of 972 atoms for the PP model. Due to the presence of a large number of atoms^38^ the cut-off energy of 520 eV was used and electronic integration over the Brillouin-zone was carried out using a 2 × 2 × 2 Monkhorst–Pack-mesh for the PE model and 1 × 1 × 1 Monkhorst–Pack-mesh for the PP model. The van der Waals interaction plays a key role in describing the interactions between polymer chains. The van der Waals-density functional (vdW-DF) method proposed by Dion et al.^39^ was adopted so that the lattice constant of the crystalline polymer model could be predicted accurately. Considering the model of lithium ion intercalated polymer crystal, charge correction is required for the measurement of total energy due to the slower convergence with respect to supercell volume. The corrections were made for finite-size effects according to Freysoldt et al.^40^. In order to let the charged system to have good convergence efficiency, cubic cell was maintained during the relaxation. In addition, the total energy of molecules was also calculated by VASP. Because of the periodic boundary condition used in VASP, it takes large cell size to prevent interference between the images of the model. In this study, the individual cell size was tested for Li^+^, PF_6_^−^, PF_5_, 3EC + Li^+^, LiF, LiPF_6_, EC, 4EC + Li^+^ to accurately calculate the converged energy. This study also considered the effect of solvent. Mathew et al.’s implicit solvation model^41^ was used. This effective method is an appropriate solution to access the solvation energies of molecular system. The implicit solvent dielectric constant was 31.9 to mimic the EC and DEC mixture (1:2 in molar ratio). The model of molecules and crystalline PE and PP was established by GaussView and VESTA^42^, respectively. The simulated XRD pattern was calculated using VESTA software.

## Supplementary Information

Supplementary Information.
