# Supplementary material for: Preferential lattice expansion of polypropylene in a trilayer polypropylene/polyethylene/polypropylene microporous separator in Li-ion batteries
Source: Sci Rep. 2021 Jan 21;11:1929. doi: 10.1038/s41598-021-81644-3 (PMC7820226; doi:10.1038/s41598-021-81644-3)
Supplement: Supplementary file 1 — Supplementary Information. [file 41598_2021_81644_MOESM1_ESM.docx]

**Electronic Supporting Information**

**Preferential Lattice Expansion of Polypropylene in a Trilayer Polypropylene/Polyethylene/Polypropylene Microporous Separator in Li-ion Batteries**

Wen-Dung Hsu^1,2^, Po-Wei Yang^3^, Hung-Yuan Chen^4^, Po-Hsien Wu^1^, Pin-Chin Wu^5^, Chih-Wei Hu^3^, Lakshmanan Saravanan^4^, Yen-Fa Liao^6^, Yen-Teng Su^7^, Dinesh Bhalothia^3^, Tsan-Yao Chen^2,3*^, Chia-Chin Chang^2,4,5*^

^1^Department of M*aterials Science and Engineering, National Cheng Kung University, Tainan 70101, Taiwan*

*^2^Hierarchical Green-Energy Materials Research Center, National Cheng Kung University, Tainan 70101, Taiwan*

*^3^Department of Engineering and System Science, National Tsing-Hua University, Hsinchu 300, Taiwan*

*^4^R & D Center for Li-ion Battery, National University of Tainan, Tainan 70005, Taiwan*

*^5^Department of Greenenergy, National University of Tainan, Tainan 70005, Taiwan*

*^6^National Synchrotron Radiation Research Center, 101 Hsin-Ann Road, Hsinchu, 30013, Taiwan.*

*^7^BenQ Materials Corporation, Guishan, Taoyuan 33341, Taiwan*

*Corresponding authors:

Dr. Chia-Chin Chang

Department of Greenergy

National University of Tainan,

Tainan 700, Taiwan

Email: ccchang@mail.nutn.edu.tw

Tel: +886-6-2144736

Fax: +886-6-2602205

Dr. Tsan-Yao Chen

Department of Engineering and System Science,

National Tsing-Hua University,

Hsinchu 300, Taiwan

Email: chencaeser@gmail.com;

Tel: +886-3-5715131#34271.


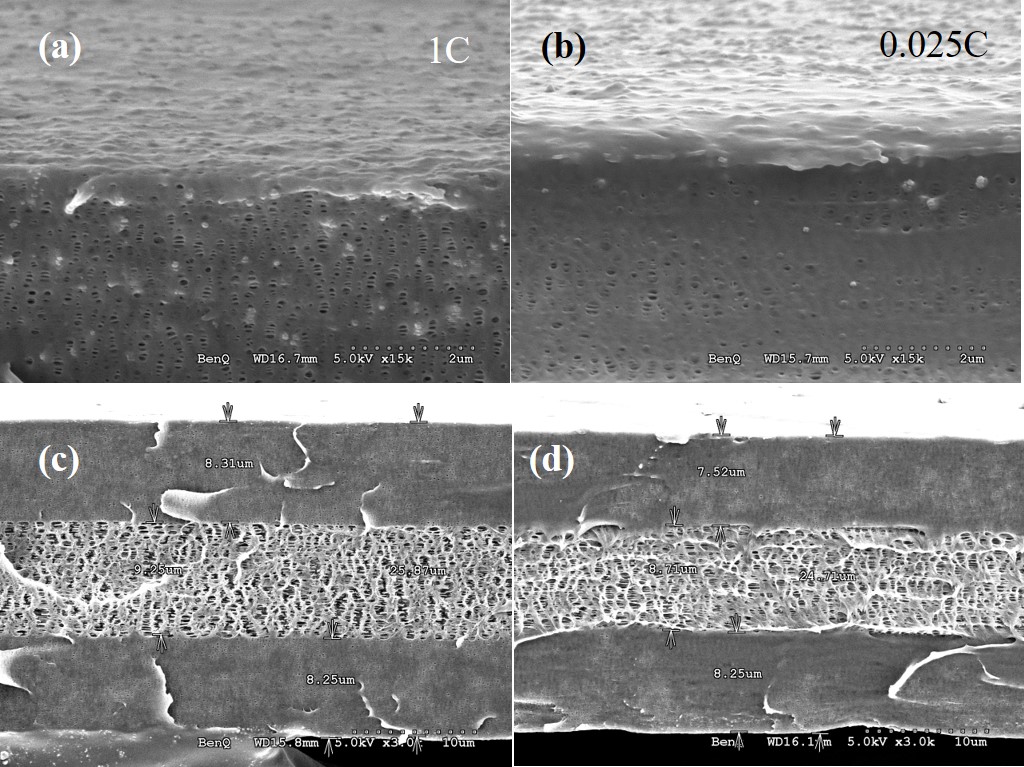


**Fig. S1.** High-magnified cross-sectional SEM images of trilayered PP/PE/PP separator. (a), (b) top PP layer, (c) after the rate rest of high (1.0 C) rate 5 cycles and (d) after the rate rest of low (0.025C) rate 5 cycles.


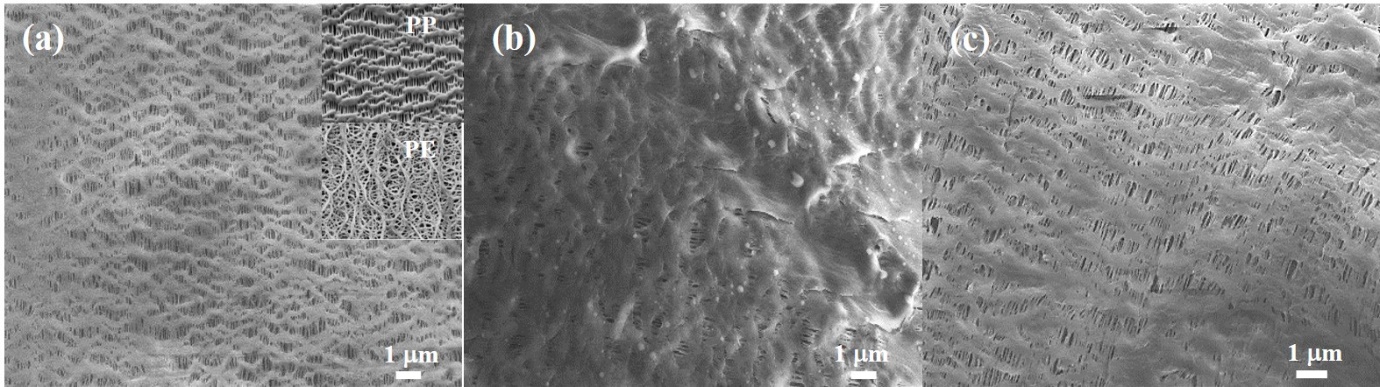


**Fig. S2.** SEM images of trilayered PP/PE/PP separator. (a) Fresh, (b) and (c) after charged at current density of 0.75 mA cm^-2^g^-1^ and 1.27 mA cm^-2^g^-1^, respectively.


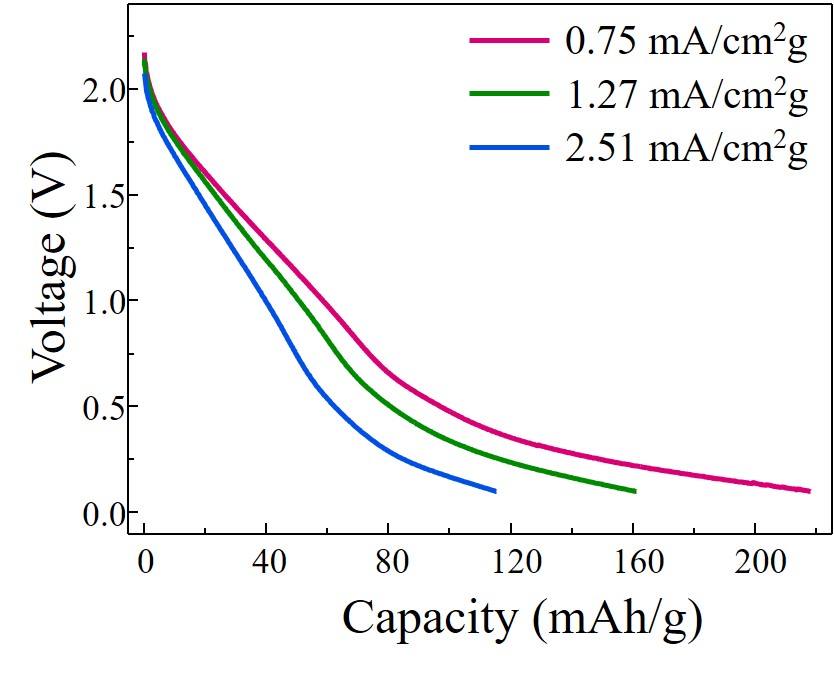


**Fig. S3.** Plot of voltage versus capacity of charging with three different C-rate (0.75, 1.27 and 2.51 mA cm^-2^g^-1^)


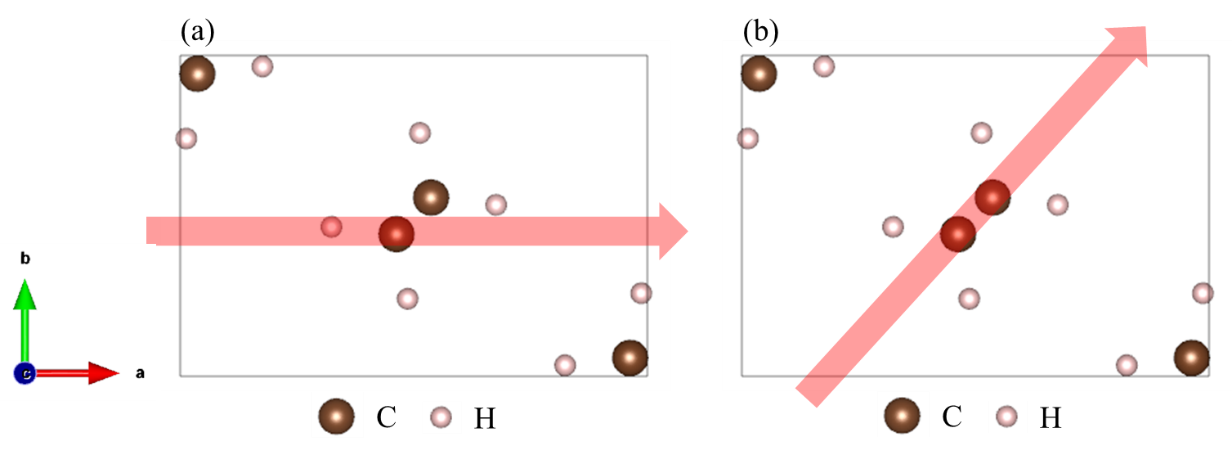


**Fig. S4.** Expansion of PE along the vertical direction of crystalline planes. (a) (200) plane and (b) (110) plane.


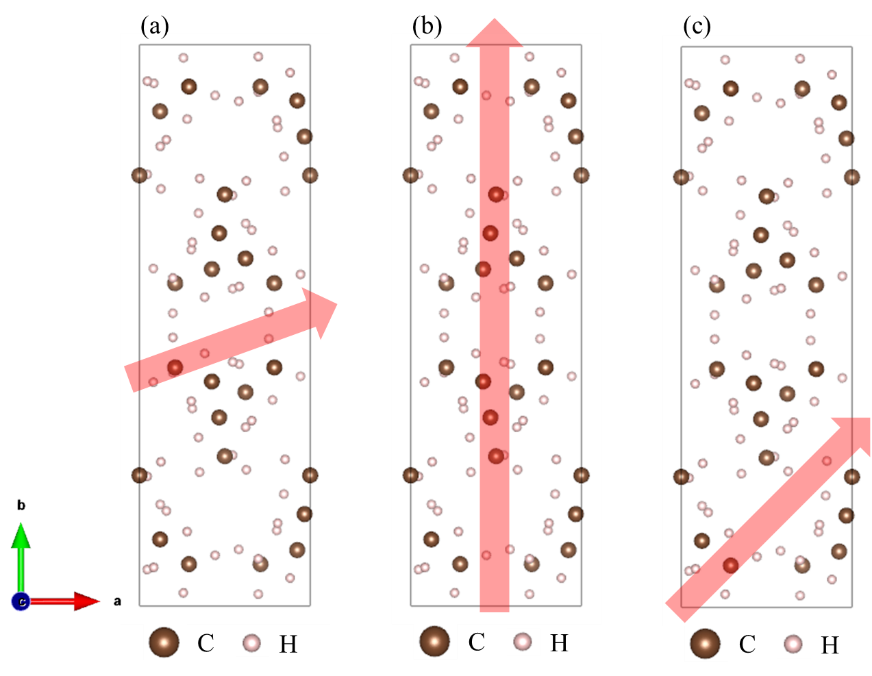


**Fig. S5.** Expansion of PP along the vertical direction of crystalline planes. (a) (110) plane (b) (040) plane and (c) (130) plane.

(a)


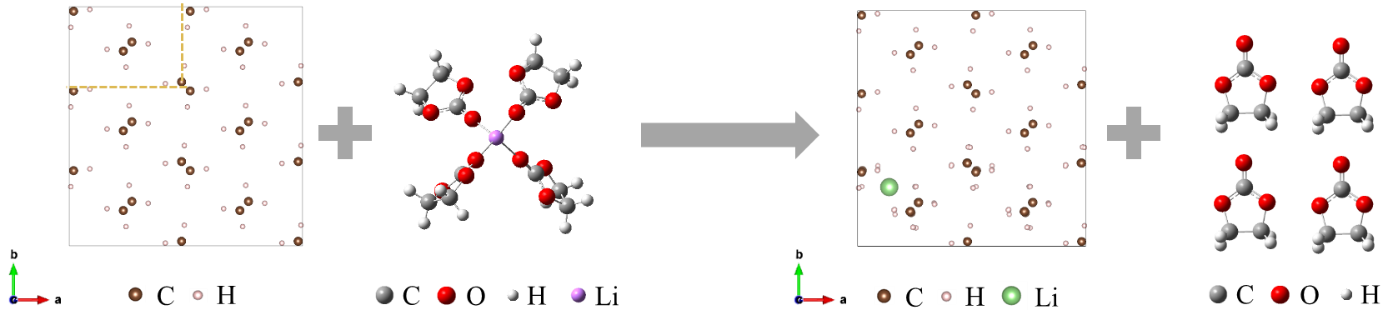


(b)


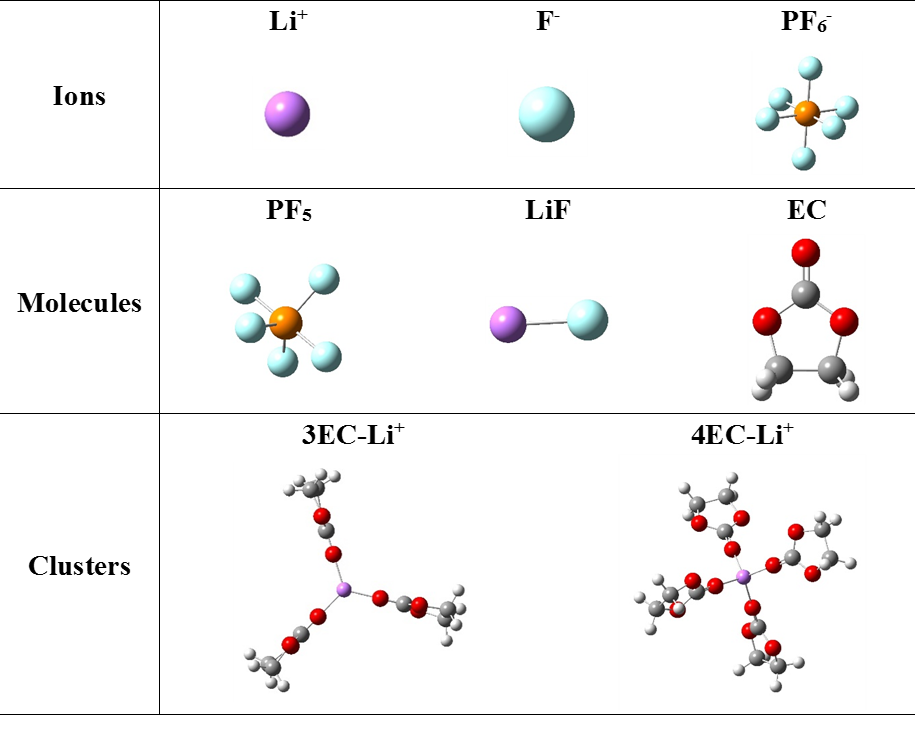


**Fig. S6.** (a) Diagram to illustrate the calculation of reaction energy of Li-ion insertion into PE crystal as given in Eq. (2). The grey boxes show the boundaries of the PE crystal model that is periodically repeated in three dimensions. (b) The models of ions, molecules and clusters used in the reaction energy calculations.

Figure S7 Brunauer–Emmett–Teller (BET) sorption curves of PP/PE/PP separators in pristine state and after cycle test at 0.025, 0.1 C rates.

Table S 1 Brunauer–Emmett–Teller (BET) test determined surface area of PP/PE/PP separators in pristine state and after cycle test at 0.025, 0.1 C rates.

| Sample | surface area (m^2^ g^-1^) |
| --- | --- |
| Pristine | 39.31 |
| 0.025C | 42.72 |
| 1.0C | 30.9 |
